# Lysosomal Ceramide Metabolism Disorders: Implications in Parkinson’s Disease

**DOI:** 10.3390/jcm9020594

**Published:** 2020-02-21

**Authors:** Silvia Paciotti, Elisabetta Albi, Lucilla Parnetti, Tommaso Beccari

**Affiliations:** 1 Laboratory of Clinical Neurochemistry, Department of Medicine, University of Perugia, Sant’Andrea delle Fratte, 06132 Perugia, Italy; silvia.paciotti@unipg.it (S.P.); lucilla.parnetti@unipg.it (L.P.); 2 Section of Physiology and Biochemistry, Department of Experimental Medicine, University of Perugia, Sant’Andrea delle Fratte, 06132 Perugia, Italy; 3 Department of Pharmaceutical Sciences, University of Perugia, Via Fabretti, 06123 Perugia, Italy; elisabetta.albi@unipg.it

**Keywords:** ceramide metabolism, Parkinson’s disease, α-synuclein, GBA, GLA, HEX A-B, GALC, ASAH1, SMPD1, ARSA

## Abstract

Ceramides are a family of bioactive lipids belonging to the class of sphingolipids. Sphingolipidoses are a group of inherited genetic diseases characterized by the unmetabolized sphingolipids and the consequent reduction of ceramide pool in lysosomes. Sphingolipidoses include several disorders as Sandhoff disease, Fabry disease, Gaucher disease, metachromatic leukodystrophy, Krabbe disease, Niemann Pick disease, Farber disease, and GM2 gangliosidosis. In sphingolipidosis, lysosomal lipid storage occurs in both the central nervous system and visceral tissues, and central nervous system pathology is a common hallmark for all of them. Parkinson’s disease, the most common neurodegenerative movement disorder, is characterized by the accumulation and aggregation of misfolded α-synuclein that seem associated to some lysosomal disorders, in particular Gaucher disease. This review provides evidence into the role of ceramide metabolism in the pathophysiology of lysosomes, highlighting the more recent findings on its involvement in Parkinson’s disease.

*  Correspondence tommaso.beccari@unipg.it

## 1. Introduction

Ceramides (Cers) are a family of bioactive lipids belonging to the class of sphingolipids (SphLs). Cers differ in their fatty acid composition (medium chain (C12–C14), long chain (C16–C18), very-long chain (C20–C24), and ultra-long chain (≥C26) and in their saturation state. In the past, Cers were known only as structural lipids and advances over the past decade have opened new horizons. It has been proposed that different Cer species are involved in physiological and pathological cellular processes (e.g., inflammation [1], cell proliferation, differentiation, apoptosis, and cancer) depending on their ability to influence membrane properties and interact with effector proteins [2], and play a role in neurodegeneration [3]. Stith et al. [2] have highlighted new faces of Cers discovered thanks to the generation and characterization of KO mice for the ceramide-metabolizing enzyme, as well as for the identification of their specific inhibitors and antibodies, and to the design of synthetic Cer derivates. Thus, in the time, Cers, the enzymes for their metabolism, and consequently their derivates have been better understood as possible diagnostic markers and as drug targets for innovative therapeutic strategies. This review provides evidence into the role of ceramide metabolism in the physiopathology of lysosomes, highlighting recent findings on its involvement in Parkinson’s disease (PD).

## 2. Interplay of Ceramides and Lipid Containing Ceramides between Lysosomes and Other Cellular Compartments

**Figure 1:**
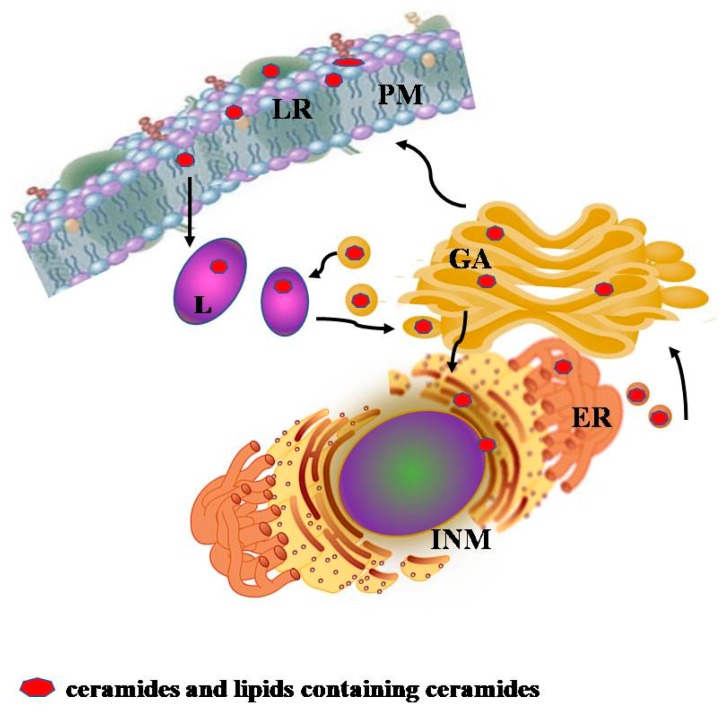
Schematic representation of the interplay between lysosomes and other cellular compartments of ceramides and lipids containing ceramides. Description in the text. ER, endoplasmic reticulum; GA, Golgi apparatus; INM, inner nuclear membrane; L, lysosome; LR, lipid raft; PM, plasma membrane.

Cers reside in different cellular compartments (Figure 1). They are synthesized in the endoplasmic reticulum and processed in the Golgi apparatus to form sphingomyelin (SM) and more complex SphLs, the glycolsphingolipids (GSLs) [4]. Cer, SM, and GSLs are then distributed in all cellular compartments with different specific destinies and roles. In the plasma membrane, Cers are both randomly distributed and located in specific structures called lipid rafts [5]. They are responsible for the normally existing lipid asymmetry by influencing the mechanical stability of the membrane [4], and for the structure and regulation of lipid rafts [6]. In the inner nuclear membrane, they are concentrated in lipid microdomains, where they regulate the active chromatin function [7]. Cellular membrane Cer can derive from SM directly in loco but also from SM and GSLs after a more complex mechanism of degradation. In fact, while proteins, glycoproteins, or oligosaccharides can be degraded by soluble enzymes in different cell sites, the degradation of membrane SM and GSLs requires a much more complex catabolic system that occurs in lysosomes [8,9]. Thus, membrane fractions, containing SM and GSLs, reach lysosomes by the endocytic vesicular flow, through the early and late endosome from the Golgi apparatus [10]. After reaching the lysosomes, Cers produced from SM and GSL degradation, and metabolism intermediates are continuously recycled and re-utilized in salvage processes [10,11]. In particular, Cer, fatty acids, and sugar are exported from the lysosomes through specific membrane proteins and reach again the Golgi apparatus where they are recycled into the biosynthetic pathway [11]. Even if very little is known about this mechanism of transport, it is well known as an interplay between lysosomes and plasma membrane/ cellular organelles by processes of membrane fusions, which could be responsible for the shift of GSL from one cellular compartment to another. Ultrastructural examination of cells, from patients with defects of GSL degradation, highlighted accumulation of non-degradable lipids in multi-vesicular storage bodies, that are responsible for different diseases in relation with the implicated enzymes.

## 3. Ceramide Metabolism in Cell Membranes

**Figure 2:**
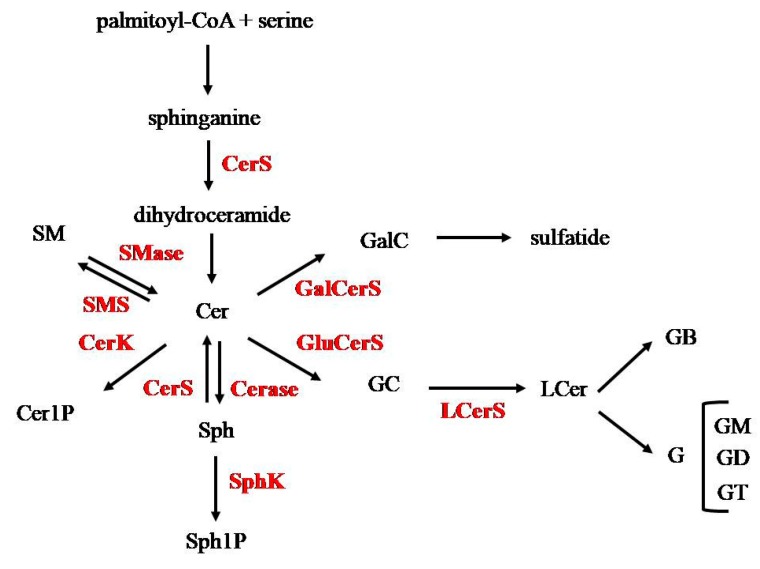
Ceramide production and utilization. In yellow, simple sphingolipids (SphLs); in green, glycosphingolipids (GSLs); in red, enzymes. Description in the text. Cer, ceramide; Cer1P, ceramide-1-phosphate; CerS, ceramide-synthase; CerK, ceramide-kinase; G, ganglioside; Cerase, ceramidase; GalC, galactocerebroside; GalCerS, galactosylceramide-synthase; GB, globoside; GC, glucocerebroside; GD, disialoganglioside; GluCerS, glucosylceramide-synthase; GM, monosialoganglioside; GT, trisialoganglioside; LCer, lactosylceramide; LCerS, lactosylceramide- synthase; SM, sphingomyelin; SMase, sphingomyelinase; SMS, sphingomyelin-synthase; Sph, sphingosine; Sph1P, sphingosine-1-phosphate; SphK, sphingosine-kinase.

Cer is the heart of SphL metabolism. It is produced in cells through three pathways: de novo synthesis, sphingosine (Sph) reacylation (salvage pathway), and breakdown of SM [12] and it is used to form other simple SphLs as SM, Cer-1-phosphate (Cer1P), Sph, and consequently, Sph-1-phosphate (S1P) and GSLs (Figure 2). The novo synthesis starts from the condensation of an activated fatty acid (palmitoyl-CoA) and an amino acid (serine) by serine palmitoyl-CoA acyltransferase to form 3-dehydro-D-sphinganine that is reduced to sphinganine (also called dihydrosphingosine) and acetylated to N-acylsphinganine (also called dihydroceramide) by Cer synthase (CerS) and finally reduced to Cer. The breakdown of Cer by ceramidase (Cerase) results in the formation of Sph [13], that can be phosphorylated to S1P by Sph-kinase (SphK) or can be acylated with a fatty acid to form newly Cer by CerS. Therefore, CerS, a family of six enzymes located primarily in the endolasmic reticulum, acts by acetylating both N-acylsphinganine and Sph by activating the Sph salvage pathway. The recycling of Sph covers up to 80% of the need of the long chain base for SphL biosynthesis [11].

Moreover, Cer can derive from SM for the action of sphingomyelinase (SMase), by producing free Cer and phosphocholine. SMase is a family of enzymes that are named on the basis of their optimal pH activity as acid, neutral, and alkaline SMase and are located in different subcellular compartments [14]. On the other hand, Cer can be used to resynthesize SM by SM-synthase (SMS) that removes sphosphocholine from phosphatidylcholine (PC). 

Cer is also the backbone to synthesize GSLs, consisting of cerebrosides (Crbs), the simplest GSLs, sulfatides characterized by the presence in the molecule of a sulphate group, globosides (GBs), and gangliosides (Gs), the most complex GSLs. In Crb, Cer is linked to a single monosaccharide commonly galactose (galactocerebroside, GalC) or glucose (glucocerebroside, GC) synthesized by galactosylceramide-synthase (GalCerS) and glucosylceramide-synthase (GluCerS), respectively. GalC is used to synthesize globoside (GB). The action of lactosylceramide-synthase (LCerS) on GC results in the formation of lactosylceramide (LCer). The addition of a sugar next to LCer is responsible for the GB and G. InGs, Cer is linked to a branched oligosaccharidic chain constituted by glucose, galactose, *N*-acetylgalactosamine (GalNAc), and sialic acid. The major form of sialic acid is N-acetylneuraminic acid (NANA). Gs are classified according to the number of sialic acid residues (from 0 to 3). Monosialogangliosides such as GM1, GM2, or GM3 are usually considered as simple gangliosides, whereas disialo-gangliosides GD3, GD2, GD1b, and trisialo-ganglioside GT1 bare more complex gangliosides. Among over 60 known natural Gs, monosialo-tetrahexosyl-ganglioside (GM1), GD1a, and GD1b are the most common ones [15].

## 4. Ceramide Metabolism in Lysosomes and Sphingolipidoses

The interest of ceramide metabolism in lysosomes goes back to more than 50 years ago when De Duve and Wattiaux [16] had recognized that several lysosomal diseases arise from deficiencies in enzymes involved in the breakdown of simple and complex SphLs. Subsequently, Weinreb et al. [17] identified the highest specific activity of acid SMase (aSMase), glucocerebrosidase (GBA), and galactocerbrosidase (GALC) in lysosomes. Therefore, it was evident since those times that the lysosomal Cer can derive from the degradation of simple Sphs, such as SM, or complex SphLs, such as GSLs. The research of those years represented the basis for understanding the role of SphLs in health and disease. Therefore, the knowledge of biology and functionality of lysosomes related to Sphs metabolism is fundamental for cell physiology, considering that lysosomal digestion of membranes is essential for cellular membrane homeostasis [11]. Metabolism of ceramide in lysosome and related pathologies has long been an interesting topic for many researchers.

**Figure 3:**
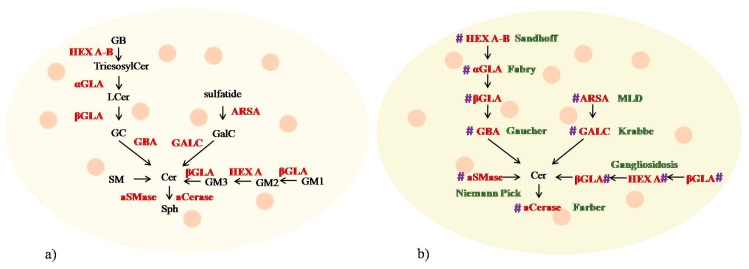
Ceramide metabolism in lysosome and sphigolipidoses. (**a**) ceramide production and utilization; (**b**) gene defects and pathological disorders. Enzymes are reported in red and diseases in green. Description in the text. aCerase, acid ceramidase; aSMase, acid sphingomyelinase; ARSA, arylsulfatase; Cer, ceramide; GB, globoside; GC, glucocerebroside; GalC, galactocerebroside; GALC, galactocerebrosidase; GBA, glucocerebrosidase; GLA, galactosidase; GM, monosialoganglioside; Hexa, hexosoaminidase; LCer, lactosylceramide; SM, sphingomyelin; Sph, sphingosine.

Lysosomal Cer can be produced (Figure 3a): (1) from the breakdown of GB by hexosaminidase A and B (HEX A-B) to form trihexosylceramide that loses one unit of galactose thanks to the α-galactosidase (GLA) to produce LCer. LCerloses, another unit of galactose by β-GLA to form GC that, after the release of a glucose molecule, generates Cer; (2) from sulfatide that loses a sulfate group by arylsulfatase A (ARSA) to form GalC from which GALC removes one unit of galactose to produce ceramide; (3) from GM1 that loses one unit of galactose thanks to β-GLA to form GM2 that, in turn, loses GalNac by HEX A and forms GM3. GM3 after the release of NANA by β-GLA, produces LCer that loses initially one unit of galactose by β-GLA and forms GC, and then one unit of glucose by GBA to produce ceramide; (4) from the SM breakdown by aSMase with the release of phosphocholine. 

Lysosomal Cer can be degraded to Sph by acid Ceramidase (aCerase) (Figure 3a).

Over time, different inherited genetic diseases characterized by the unmetabolized GSLs, and consequently, by the reduction of ceramide pool in lysosomes, called sphingolipidoses (SPs), have been discovered (Figure 3b). SPs belong to a wider class of monogenic disorders known as lysosomal storage diseases (LSDs). Over 60 LSDs are known and they are classified on the basis of the biochemical nature of the accumulating substrate, i.e., SPs, mucopolysaccharidoses, and oligosaccharidosis. However, despite the distinct storage materials in different LSDs, many biochemical, cellular, and clinical features are common [18]. Ever more specific research is aimed at identifying the distinctive aspects of LSDs. Among LSDs, SPs are the main lysosomal disorders to which scientific research has paid particular attention in order to develop new therapeutic strategies. SPs include Sandhoff disease, Fabry disease, Gaucher disease, metachromatic leukodystrophy (MLD), Krabbe disease, Niemann Pick disease, Farber disease, and gangliosidosis, which are characterized by the gene defect in different enzymes, as reported in Figure 3b.

In SPs, lysosomal lipid storage occurs in both the central nervous system (CNS) and visceral tissues. CNS pathology is a common hallmark of SPs, and although SPs can be mainly considered pediatric disorders, an increasing number of evidences have demonstrated that the impaired activity of enzymes involved in SphL catabolism is associated to the development of neurodegenerative diseases characteristic of adulthood, like PD. 

PD is the most common neurodegenerative movement disorder, characterized by the accumulation and aggregation of misfolded α-synuclein (α-syn) in large cellular structures named Lewy bodies (LBs), that represent the pathological hallmark of several genetic and sporadic forms of PD [19]. Several studies have highlighted that changes in the SPs pattern (particularly in the sphingolipid metabolites that accumulate in LSDs such as glucosylceramide, psychosine, glucosylsphingosine, and gangliosides) can influence α-syn homeostasis causing its accumulation and aggregation into the cells.

## 5. Ceramide Metabolism Monogenic Disorders and Parkinson’s Disease

### 5.1. GBA 

GBA, a lysosomal membrane protein, is encoded by a single gene (*GBA*) located on chromosome 1 [1]. GBA catalyzes the hydrolysis of GlcCer into free ceramide and glucose [2,3]. Thereby, it plays a central role in the degradation of complex lipids and the turnover of cellular membranes [4]. Furthermore, through the production of ceramides, GBA participates to the PKC-activated salvage pathway of ceramide formation [5]. The activity of GBA depends on many factors such as proper folding and lysosomal localization, which are influenced by mutations in the GBA encoding gene, and are regulated by various GBA-binding partners including Saposin C, progranulin, and heat shock proteins [6]. 

More than 400 mutations on *GBA* gene have been described; most of them lead to the synthesis of GBA with decreased catalytic function and/or stability (The Human Gene Mutation Database: http://www.hgmd.cf.ac.uk/ac/index.php). Deficiency of GBA activity causes Gaucher disease (GD), the most common LSD. Three types of GD reflecting the degree of GBA deficiency are known. Type 1, the most common form, that occurs with a prevalence of 1:50,000–1:100,000 in the general population and of 1:855 in the Ashkenazi Jewish [7,8], is distinguished from types 2 and 3 by the lack of primary CNS involvement. In people affected by the type 1 GD, the onset can range from childhood to adulthood. Type 2 GD, the acute neuronopathic form, has an early onset with severe CNS involvement and death usually within the first 2 years of life. Patients with type 3 (subacute neuropathic) GD have neurologic symptoms with a later onset and a more chronic course than that observed in type 2 disease. The median age of onset is one year, with considerable variability.

Recently, GD has been associated with parkinsonism. The occurrence of Parkinson’s syndrome in type 1 GD was described for the first time in 1996 in a small group of GD patients who showed Parkinsonian-like symptoms [9]. Subsequently, other studies from around the world have supported clinical, neuropathological, and genetic associations between GD and PD [10,11,12,13]. In 2009, a large multicenter analysis, on more than 5000 PD patients and healthy controls showed that mutations on the *GBA* gene are the most common genetic risk factors for PD [14]. *GBA* mutations account for 10%–15% of PD patients and this percentage increases to 25% in the Ashkenazi Jewish population [15,16]. Although *GBA* mutation carriers have 5–6 fold increased risk to develop PD [14], the penetrance of PD in these people is low (from 7.6% at 50 years, to 29.7% at 80 years), thus suggesting that there are other genetic or environmental factors involved in this neurodegenerative process [17].

PD patients carrying *GBA* mutations seems clinically indistinguishable from sporadic PD, however, several studies have reported that the presence of *GBA* variants leads to earlier age of onset, higher risk of cognitive impairment, and accelerated disease progression [17,18,19,20,21,22]. It is worth to note that patients carrying the more severe mutations, such as the c.84dupG, D409H, or L444P, are more likely to develop PD (OR = 10.3) than carriers of N370S or R496H (OR = 2.3). Of interest, the T369M and E326K variants, which are not associated to GD even in homozygous state, are associated to PD with an OR of 1.78 and 1.99, respectively [23,24,25]. Furthermore, mutations associated to the neuropathic form of GD are related to more severe motor phenotypes and increased risk of non motor manifestations, including hyposmia, cognitive impairment, and dementia [24,26,27]. 

To date, the mechanism by which mutated GBA leads to neurodegeneration has not been elucidated; however, data supporting a loss of GBA function as well as a gain of toxic function mechanisms have been published. Several studies carried out on cellular and animal models of both GD and PD, as well as analysis in different cohorts of patients affected by PD, have highlighted the existence of a vicious cycle between GBA dysfunction and α-syn accumulation [16]. In brain tissues of GD patients, immunohistochemical analysis revealed the presence of aggregated α-syn and LBs [28]. Furthermore, in red blood cells of GD patients, a positive correlation between the α-syn dimer/monomer ratio and the levels of GC and the GC/Cer ratio was found [29]. There are evidences that GC and glucosylsphyngosine stabilize the toxic α-syn oligomeric intermediates, which in turn, promote the formation of α-syn aggregates [27,29,30]. Accumulated α-syn was also found in the brain of GD mouse models [31,32] and in *GBA* mutant derived dopaminergic neurons [33]. Of interest, in models of GBA deficiency, the restoring of its activity counteracts α-syn accumulation and toxicity [32,33,34]. Yang and colleagues [33] found that the activity of the lysosomal aspartyl protease cathepsin D (CatD), which is responsible for α-syn degradation, dependents on GBA activity. Particularly, they found that *GBA* mutations lead to lower levels of CatD protein and activity, and higher levels of monomeric α-syn. Restoring of GBA activity in *GBA* mutant neurons recovered CatD protein levels and activity, and led to decreased monomeric α-syn levels. It is known that Cer binds CatD and triggers its cleavage to the catalytic active form [35,36], thus it is possible that the lower levels of Cer in the lysosomal compartment due to GBA impairment, influence the CatD maturation process, leading to a decreased capacity of the lysosome to degrade α-syn.

The contribution of a gain of GBA toxic function in the development of PD has been extensively demonstrated by Maor et al. These authors found the activation of the unfolded protein response (UPR) and the development of parkinsonian signs, with death of dopaminergic cells, defective locomotion, and a shorter life span, in models of Drosophila melanogaster expressing mutant misfolded GBA [37,38,39]. The abnormal conformation of mutated GBA might lead to endoplasmic reticulum stress and lysosomal dysfunction, overwhelming the ubiquitin-proteasome pathway and causing impairment of autophagy [40]. Of interesting, when these flies were treated with the pharmacological chaperone ambroxol, which binds and removes the misfolded GBA from the endoplasmic reticulum (ER), the UPR and parkinsonian signs were partially rescued, suggesting that the signs observed in these models are a consequence of the dominant deleterious effect of the mutant misfolded GBA, rather than the result of loss-of-function. This hypothesis is also supported by the results of a recent clinical trial (ClinicalTrials.gov identifier: NCT02941822), in which the administration of ambroxol led to the improvement of motor functions in both *GBA* mutations carriers and non-carriers PD patients [41]. 

Several studies have shown reduced GBA activity in brain tissues [7,42,43,44,45,46,47], cerebrospinal fluid (CSF) [48], and dried blood spots [49] of both PD patients carrying *GBA* mutations and sporadic PD patients [50]. Particularly, GBA activity was found to be significantly reduced in cerebellum, amygdala, putamen, and, in a major extent, in the substantia nigra of PD patients carrying *GBA* mutations [42]. Murphy et al. found reduced GBA activity in the lysosomal-enriched protein fractions of early stage PD patients, more specifically in the anterior cingulated cortex [43]. Of importance, sporadic PD patients also showed significant reduction of GBA activity in the cerebellum, substantia nigra, and caudate, when compared to controls [42,47]. These results were confirmed in a Dutch cohort where reduced GBA activity was found in the substantia nigra of both PD and DLB patients [44]. Of interest, in brain tissue from late stages of PD patients, diminished GBA activity was selectively found with increased levels of α-syn [51]. In CSF, lowered GBA activity was repeatedly found in PD with respect to control subjects [45,46,48]. Furthermore, it has been demonstrated that as for brain tissue, also for CSF, the activity of GBA was significantly reduced even in sporadic PD patients, confirming that the reduction of GBA activity in PD is independent of the presence of mutations in the *GBA* gene. Taken together, these findings suggest that mutations on the *GBA* gene promote α-syn accumulation, however they also indicate the existence of alternative mechanisms independent of the *GBA* genotype which impair GBA activity in PD patients.

### 5.2. GLA

Lysosomal α-GLA hydrolyzes terminal α1,3- and α1,4-linked galactosyl residues from various glycoconjugates such as globotriaosylceramides and digalactosylceramides, within the lysosomes. The gene encoding for α-GLA (*GLA*) is located on the X chromosome (Xq22.1) [52]. Mutations in *GLA* gene cause deficiency of α-GLA activity and lead to develop Fabry disease. Fabry disease was described for the first time in 1898 [53], it is characterized by the progressive accumulation of globotriaosylceramide, globotriaosylsphingosine, and digalactosylceramide in cells, which alters the normal function of several cellular organelles and causes multi-systemic effects with neuronal, renal, cardiac, and cerebrovascular involvement [54]. To date, around 967 different *GLA* mutations have been described (listed on the Human Gene Mutation Database, http://www.hgmd.cf.ac.uk/); most of them have been identified only in individual families. In childhood, Fabry disease is typically characterized by lethargy, tiredness, pain, cutaneous abnormalities, changes to sensory organs, and gastrointestinal disturbances. In adulthood, people develop also lymphedema, proteinuria, and the first signs of renal, cardiac, or CNS/cerebrovascular disease, all symptoms that worsen over the time. The late onset forms of Fabry disease may present as a stroke, left ventricular hypertrophy, or renal failure [54].

Although Fabry disease was defined “rare” (with a prevalence of 1:50,000 [55]), recent newborn screenings revealed that this disorder is more common than expected (the incidence ranges from 1:1250 to 1:8454) [56,57,58,59]. The discrepancy among these data can be explained by the heterogeneity of the symptoms and the late onset of the disease (patients develop symptoms at their teenage or adulthood years), thus the use of the newborn screening for the diagnosis allowed to better identify people affected [54].

Several α-GLA animal models have been generated to better understand Fabry disease pathogenesis [60,61,62,63]. Among them, one *Gla* knock-out model, also overexpressing the Gb3 synthase, developed a significant neurological phenotype characterized by spontaneous tremors, slowed movements and gait disturbances [64]. It worthy to note that these findings are typical of patients affected by PD. As well as the animal model, also patients affected by Fabry disease showed parkinsonian symptoms. Orimo et al. [65] described a 68-year old man with a 5-year history of parkinsonism; upon autopsy, Fabry disease was diagnosed. In 2006, Buechner and colleagues [66] described a case of a patient affected by parkinsonism who had clinical findings consistent with the Fabry disease classic phenotype. 

Of interest, in peripheral blood leukocytes of sporadic PD patients, reduced α-GLA activity was found with respect to controls [67]. The same authors also showed significantly decreased α-GLA expression levels (both mRNA and protein) in leukocytes of PD patients with respect to age, and sex-matched healthy controls [68]. A study performed in a large cohort composed of *n* = 648 PD patients and *n* = 317 controls showed lower blood α-GLA activity associated with PD status [69]. α-GLA activity was also found to be significantly reduced in the temporal cortex of advanced PD patients [70] and in substantia nigra of sporadic PD patients [7]. α-GLA mRNA and protein (particularly the 46 kDa “active” isoform) levels were also reduced in the temporal cortex of these patients. Interestingly, negative correlations between α-GLA activity and the 17 kDa phosphorylated α-syn (p129S-α-syn) and total α-syn monomer were described. This finding is in line with the presence of phosphorylated α-syn found in the pons of a Fabry mouse model [71].

Recently, a survey study was also performed to estimate the prevalence of PD in Fabry patients and families [69]. It revealed that the prevalence of PD among the patients affected by Fabry is higher than in normal population. However, PD diagnosis was not clinically confirmed in this study, for this reason, the results should be interpreted with caution.

### 5.3. HEX A and B 

β-Hexosaminidase (β-HEX) of human tissues and body fluids exists in two main forms, HEXA and HEXB [72]. In addition, two or more forms have charged characteristics that cause them to be eluted between HEXA and HEXB on DEAE-cellulose chromatography and hence are rather loosely referred to as ‘intermediate forms’[73]. The subunit compositions of the A and B forms from human placenta are αββ and 2xββ, respectively. Presence of α-subunit of β-HEX confers the ability to hydrolyze the ganglioside GM2 and also the synthetic substrate 4-methylumbeliferyl β-N-acetylglucosamide [74]. In vivo, the GM2 activator protein is an essential component for the degradation of GM2 ganglioside by HEXA. The α and β subunits are encoded by two different genes, *HEXA* and *HEXB*, located in two different chromosomes [75]. Mutation of *HEXA* gene causes Tay-Sachs disease, a lysosomal storage disorder, also called GM2 gangliosidosis, that results from an inability to hydrolyze GM2 ganglioside. Mutations in the human GM2 activator gene cause the AB variant of GM2-gangliosidosis, a condition that is clinically indistinguishable from Tay-Sachs disease. Tay-Sachs is a neurodegenerative disease that progressively destroys neurons in the CNS. Mutations in the *HEXB* gene cause Sandhoff disease, another variant of GM2 gangliosidosis, that similarly to Tay-Sachs, causes neurodegeneration. 

There are no evidences of a link between HEXB deficiency and PD, however, several case reports describe the manifestation of parkinsonian motor symptoms in patients with either childhood- or adult-onset Sandhoff disease [76,77]. Furthermore, mutations in *HEXB* were recently confirmed as LSD gene variants in PD patients [78]. *HexB* knockout mice manifest α-syn pathology in the substantia nigra, along with GM2 ganglioside accumulation [79]. Similar traits were observed in the brain of Sandhoff disease patients in which α-syn deposits can be observed [80]. The main hypothesis of this feature is that α-syn accumulates as a consequence of the direct interaction between α-syn and GM2.

Deficiency in HEXA activity was also found to cause parkinsonian-like symptoms in patients affected by GM2 gangliosidoses [81,82], however the number of cases described is too low to hypothesize the involvement of this enzyme in PD pathology.

Increased β-HEX activity, associated to a reduced GBA activity, has been observed in CSF from sporadic PD patients and *GBA* mutation carrier PD patients [46]. A similar result was also observed in fibroblasts of PD patients with and without *GBA* mutations [83]. Conversely, in leukocyte of patients affected by PD and other genetic parkinsonism, β-HEX and GBA activities were similar between patients and controls [84]. Recently, reduced β-HEX activity has been found in the substantia nigra of sporadic PD patients compared to age matched controls [7].

More in depth investigations are required to better understand if this enzyme is involved in PD pathogenesis. 

### 5.4. GALC

GALC catalyzes the hydrolysis of different substrates including galactosylceramide (GalC) and galactosylsphingosine (also called psychosines).

Mutations in the *GALC* encoding gene cause Krabbe disease (KD), also known as globoid cell leukodystrophy (GLD). KD is characterized by the decrease of GALC activity and the impaired degradation of GalC and psychosines in the peripheral and CNS [85]. To date, more than 130 mutations on the *GALC* encoding gene have been recognized, and among them, at least 128 have been reported as the cause of KD [86]. It is worth to note that the presence of polymorphisms in the *GALC* gene resulting in amino acid substitutions can generate GALC proteins enzymatically less active, this feature explains the relatively broad range of GALC activities observed also in the healthy population [87].

KD typically presents with infantile onset in the first 6 months of life; patients show a rapid and progressive course characterized by irritability, hypersensitivity to external stimuli, severe mental and motor deterioration, and death by age 2 [88,89]. However, late-onset forms and adult form of KD have been recognized. In particular, patients affected by the adult form show a milder and heterogeneous phenotype, with a slower rate of progression and a normal lifespan [90].

The decrease of GALC activity in KD patients causes the accumulation of GalC in cerebral macrophages, and psychosines. To date, there is no experimental evidence of the toxic effects due to GalC accumulation whereas, the increase of psychosines concentration has been shown to be neurotoxic to oligodendrocytes and Schwann cells leading to central and/or peripheral demyelination [90]. Psychosines are cationic lipid, generally present at low concentration in the brain of healthy people. The increase of these lipids in KD patients induces an inflammatory response, which is the main cause of cell death in Krabbe brain tissue [91]. Psychosines accumulation also leads to mitochondrial dysfunctions and interferes with cell signaling due to the disruption of lipids raft domains [92]. It was been found that psychosines also facilitate α-syn fibrillization [93]. α-syn pathology was observed in brain of subjects carrying mutations or pathogenic variants on the GALC encoding gene. Smith and colleagues [93] showed that galactosylsphingosine accelerates aggregation of α-syn in a dose-dependent manner, this is interesting taking into account that in PD patients, higher levels of galactosylsphingosine were found with respect to controls [94].

In the brain of the Twitcher mouse (the mouse model of KD), α-syn aggregation was observed, further underlining a link between KD and late onset synucleinopathies [94]. It is worth to note that similarly to LB accumulation in PD [95], in these mice, neuronal aggregates of α-syn originate in the hindbrain’s medulla and pontine regions and then spread rostrally and dorsally into the midbrain structures, affecting the cerebral cortex in only the late stages of the disease. 

Increased plasma oligomeric α-syn levels were also found in a small group of patients affected by KD [95].

Furthermore, a large genome-wide association study meta-analysis showed the correlation between some genetic variants of *GALC* and a higher risk to develop PD [78]. Recently, similar data were observed in a small Chinese cohort composed of 250 PD patients and 240 healthy controls, in which the common polymorphism rs8005172 of *GALC* was found to be associated with the late onset of PD. As for other lysosomal enzymes, GALC activity is also reduced in aging. Thus, it is possible that the partial loss of GALC activity due to the aging, in combination with other factors, can lead to the neurodegeneration observed in PD.

### 5.5. Acid Sphingomyelinase

aSMase is a lysosomal enzyme able to hydrolyze SM to produce Cer. It differs from other SMases for both chemical–physical characteristics and functions [96]. aSMase was involved in the response to stress when aSMase rapidly translocates from lysosomes to the outer leaflet of the plasma membrane where degrades SM, in cancer [97,98], and in the physiopathology of the CNS. In aging, aSMase derived from endothelial cells plays a critical role in blood–brain barrier disruption by causing damage to the caveolae–cytoskeleton via protein phosphatase 1-mediated ezrin/radixin/moesindephosphorylation [99]. The authors showed data on mice overexpressing brain endothelial cell-specific aSMase that presented acceleration of blood–brain barrier impairment and neuronal dysfunction. A local production of Cer by aSMase as well as by nSMase was found in the corona of the senile plaques [100]. Many observations have been reported on CNS pathology. In fact, aSMase activity was increased in ischemic stroke [101], in autoimmune encephalomyelitis [102], and in astrocytes and oligodendrocytesduring multiple sclerosis [103,104]. Furthermore, Pieragostino et al. [105] demonstrated a high level of aSMase in CSF from patients affected by multiple sclerosis, showing that the activity of this enzyme was related to the number of exosomes and to the content of enzyme per exosome. Therefore, the authors defined exosomes as nano-carriers of aSMase and highlighted the positive relation between exosomal aSMase and severity of disease. On the other hand, deficiency of aSMase gene (*SMPD1*) enhanced myelin repair after cuprizone-induced demyelination in a mouse model used to examine myelin destruction and remyelination in multiple sclerosis. 

Additionally, aSMase has also been proposed to play a role in dementia. Sarrafpour et al. [106] performed a study in CSF from patients with late-onset Alzheimer’s disease (AD) and patients with other dementia phenotypes showing that the activity of aSMase was higher in the last patients than in AD. Fonteh et al. [107] showed that the aSMase activity was significantly reduced in CSF from AD patients compared with controls and that was independent of depression and psychotropic medications. In contrast to this, de Wit et al. [108] provided evidence of an increased level of aSMase in AD implicated in cerebral amyloid angiopathy characterized by a deposit of amyloid-β (Aβ) in cortical capillaries.

In the last years, the study of the deficiency of *SMPD1,* causing Niemann-Pick disease, has greatly expanded. Niemann-Pick disease is characterized by the accumulation of SM in lysosomes of multiple tissue and can be divided in Niemann-Pick disease “type A” (NPA) and “type B” (NPB). The forms of NPA and NPB differ from Niemann-Pick disease type C (NPC) that is caused by mutations in genes (*NPC1* and *NPC2*) encoding for proteins involved in cholesterol efflux from lysosomes [109]. Clinically, NPA patients present developmental delay, hepatosplenomegaly, and progressive neurodegeneration, and typically undergo death at 2–3 years of age. NPB patients have hepatosplenomegaly without neurological defects and usually live into adulthood [96]. McGovern et al. [110] studied the evolution of NPA disease from the neonatal period. The first sign was hepatosplenomegaly; then, the defect in the adaptive behavior and motor skills appeared after ten months and the expressive language was reached only at twelve months of age. The most important causes of death were respiratory failure and bleeding complications [110]. The brain of NPA patients was found to be atrophic, with a loss of cerebral and cerebellar cortex cells and white matter demyelination [96]. Interestingly, the hippocampus lacked of aSMase but had a normal level of the nSMase gene and enhanced protein levels probably due to the slower protein degradation rate [96]. The stimulation of nSMase activity could be useful to partially limit the damage induced by SM accumulation.

While there is a plethora of information about neurodegeneration in NPA and NPB, there is a lack of knowledge about the relationship between the gene responsible for these diseases and other neurodegenerative disorders as PD. Foo et al. [96] identified a variant (p.R591C) in the SMPD1 gene associates with PD. In time, many mutations associated with an increased risk for PD were observed in different cohorts [96,111,112,113,114]. Later, Song et al. [115] reported *SMPD1* mutations in 3.64% of Chinese PD patients that were predicted to have a damaging effect on the structure and function of the aSMase enzyme. Association between Leu-Ala (Val) repeat variants in *SMPD1* and Chinese Han patients with sporadic PD was reported [116]. A systematic analysis of whole exome sequencing dataset currently available, further confirmed the association between mutations on the *SMPD1* gene and the higher susceptibility to develop PD [78]. Moreover, reduced aSMase activity was related with a 3.8 to 5.8 earlier onset of PD and *SMPD1* knockout and knockdown resulted in increased α-syn levels in dopaminergic cells [116].

### 5.6. Acid Ceramidase

aCerase is a lysosomal enzyme able to hydrolyze Cer to produce Sph. It belongs to a family of 5 Cerase: aCerase, neutral Cerase, alkaline Cearase1, 2, and 3 encoded by five different genes (*ASAH1*, *ASAH2*, *ACER1*, *ACER2*, and *ACER3*, respectively). The Cerase are classified according to their optimal pH for catalytic activity. aCerase deficiency in humans or genetic knockout mice is not compensated for by other Cerase [117]. The enzymatic activity has fundamental roles in immune processes [118,119], in cancer [120,121,122], in diabetes, and other disease [123]. The deficiency of the gene for the aCerase synthesis, *ASAH1*, is responsible for two rare inherited disorders, Farber lipogranulomatosis (Farber disease) and spinal muscular atrophy with myoclonic epilepsy (SMA-PME) [124]. In 1947, Farber described the first case of “disseminated lipogranulomatosis” in a 14-month-old infant and he later expanded his observation by studying other small patients [125]. Farber had understood that it was a pathology of accumulation but, at that time, the Cer had not been identified yet; it would be identified later, in 1963 from a biopsy of a patient’s kidney [126]. He had noticed similarities with Niemann-Pick’s disease but there were differences that could not be explained. In those years, also the aCerase was identified. This discovery was followed by elucidation of the not detectable aCerase in post-mortem tissue from an FD patient [127].

Classical Farber disease is characterized by a triad of symptoms onset in the first weeks of life of painful, progressive deformity, palpable subcutaneous nodules in the major joints and pressure areas, and a hoarse cry resulting from granulomas of the larynx and epiglottis [128].

With the involvement of CNS, patients have significant motor and cognitive impairments leading to rapid deterioration and death. Life expectancy of children is usually less than two years. In patients with an attenuated form of Farber disease, CNS is not involved and life expectancies are longer. SMA-PME is a rare variant of childhood spinal muscular atrophy and progressive myoclonic epilepsy. In SMA, there is a defect of survival motor neuron gene 1 (*SMN1*) and in PME of missing in metastasis (*MIM*), also known as the *MTSS1* gene. The SMA-PME disease manifests around three years of life with a progressive lower motor neuron disease of proximal lower-extremity weakness, followed by progressive myoclonic and atonic seizures, tremulousness/tremor, and sensorineural hearing loss, and cognitive decline [128].

Considering the young age of the patients and the low life expectancy, there are no studies of correlations between Faber/SMA-PME disease and PD. However, burden of rare mutations in *ASAH1*, were associated with PD in a large sequencing study [78].

Of interest, a recent work showed that the inhibition of aCerase by carmofur in *GBA*-PD derived dopaminergic neurons, resulted in lower levels of α-syn [129]. It is worth to note that also WT cells treated with carmofur showed reduced α-syn levels. As discussed before, maturation and activation of CatD is dependent of Cer levels. Thus, it might be possible that the inhibition of aCerase, increases the levels of Cer and consequently CatD activity. In line with this hypothesis, there are data from Heinrich et al., who described enhanced CatD activity in cells derived from patients affected by Farber disease, whereas in aSMase deficient cells derived from NPA patients, reduced CatD activity was found.

### 5.7. ARSA

ArylsulfataseA (ARSA) degrades sulfated glycolipids; one of its major substrates is cerebroside 3-sulfate, a lipid found mainly in myelin membranes, where it accounts for 3% to 4% of total membrane lipids. The complete deficiency of ARSA activity due to homozygousor compound heterozygous mutations causes MLD, an autosomal recessive LSD, which is present in the population with a frequency of 1:40,000 [130]. Patients affected by MLD show accumulated sulfatide in all tissues, mainly in the nervous system, where it leads to progressive demyelination [131].

MLD presents three different forms: (1) a severe late-infantile form starting between the ages of 1 and 3 years; (2) a juvenile form with an age of onset at 3 to 16 years; and (3) adult forms that may not become apparent before the third decade of life. The later forms are characterized by a slow progression and patients may survive for as much as 20 years after the disease has started. Of interest, ARSA deficiency can also be observed in individuals who are clinically healthy (pseudodeficiency), who show only 10%–20% of normal enzyme activity. The frequency of ARSA pseudodeficiency allele is estimated to be between 7% and 15%, which predicts that 0.5% to 2% of the population are homozygous and thus, pseudodeficient [131]. 

Total and partial ARSA deficiency have been reported associated with movement disorders such as dystonia, chorea, athetosis, parkinsonism, or other neurological symptoms [132,133,134]. Of interest, in cerebral white matter and brain stem of MLD patients, accumulated α-syn was observed [77]. Lee et al. [135] found that *ARSA* gene deficiency leads to an increase in α-syn aggregation, secretion, and cell-to-cell propagation in vitro. Furthermore, they found that the effects on α-syn are due to a novel function of ARSA as a cytosolic molecular chaperone. This enzyme seems to interact with α-syn reducing its aggregation. It is worth to note that the binding affinity was high for the variant N352S of *ARSA*, which has been found to have a protective role in patients affected by autosomal dominant familial PD [135]. Conversely, the binding affinity of the pathogenic L300S *ARSA* was low. This very rare mutation co-segregated with PD patients with mild cognitive impairment and probable essential tremor, in one family affected by MLD. However, the association between the pathogenic mutation L300S and PD as well as the protective role of the N352S variant should be further investigated in a larger cohort to confirm these data [135].

## 6. Conclusion

In this review, we described the association between the altered ceramide catabolism and PD pathogenesis. The intracellular accumulation of α-syn associated to the death of dopaminergic neurons in the substantia nigra, is the histopathological hallmark of PD. Accumulation of α-syn is also a common feature observed in many lysosomal diseases. These findings, together with the most recent genetic studies, indicate an explicit association of variants within several lysosomal genes, including those of the ceramide catabolism, and PD.

In fact, neurodegeneration is a remarkable phenotype in nearly all lysosomal storage disorders, indicating the relevance of lysosomal degradation in maintaining neuronal health. However, the causal link between mutations on these genes and α-syn accumulation and aggregation is still unclear. Among lysosomal diseases, Gaucher disease is the most intriguing one to understand the link with Parkinson’s disease. Mutations on the GBA gene are the most significant risk factor for PD, and are the most common genetic mutation so far identified in this disease. Several studies identified a reciprocal relationship between GBA and α-syn that is common to individuals with and without GBA mutations. Many steps forward have been made to understand the relation between GBA and PD, however further studies are still needed to better piece together the mechanisms that contribute to this association and to identify other risk factors that favor the development of parkinsonism. In addition, there are already therapies available for Gaucher’s disease and new therapies are on the way, thus, these studies will be important for the discovery of more effective therapeutic approaches for PD.

The understanding of the pathophysiology of the endosomal–lysosomal–autophagic system will become of fundamental importance to develop new therapeutic strategies not only for lysosomal storage disorders but also for Parkinson’s disease.
